# "Obesity" and "Clinical Obesity" Men's understandings of obesity and its relation to the risk of diabetes: A qualitative study

**DOI:** 10.1186/1471-2458-8-311

**Published:** 2008-09-14

**Authors:** Nicola F Weaver, Louise Hayes, Nigel C Unwin, Madeleine J Murtagh

**Affiliations:** 1 Institute of Health and Society, University of Newcastle upon Tyne, Newcastle upon Tyne, UK

## Abstract

**Background:**

The 2007 Wanless report highlights the ever increasing problem of obesity and the consequent health problems. Obesity is a significant cause of diabetes. An increasing evidence base suggests that in terms of reducing diabetes and CVD risk, it is better to be "fit and fat" than unfit and of normal weight. There has been very little previous research into the understandings that men in the general population hold about the issues of weight, exercise and health; we therefore undertook this study in order to inform the process of health promotion and diabetes prevention in this group.

**Methods:**

A qualitative study in North East England General Practice using a purposive sample of men aged 25 and 45 years (selection process designed to include 'normal', 'overweight' and 'obese' men). One to one audio-recorded semi structured interviews focused on: overweight and obesity, diet, physical activity and diabetes. Transcripts were initially analysed using framework analysis. Emerging themes interlinked.

**Results:**

The men in this study (n = 17) understand the word obesity differently from the clinical definition; "obesity" was used as a description of those with fat in a central distribution, and understandings of the term commonly take into account fitness as well as weight. Men in their late 30s and early 40s described becoming more aware of health issues. Knowledge of what constitutes a 'healthy lifestyle' was generally good, but men described difficulty acting upon this knowledge for various reasons e.g. increasing responsibilities at home and at work. Knowledge of diabetes and the link between obesity and diabetes was poor.

**Conclusion:**

Men in this study had a complex understanding of the interlinked importance of weight and fitness in relation to health. Obesity is understood as a description of people with centrally distributed fat, in association with low fitness levels. There is a need to increase understanding of the causes and consequences of diabetes. Discussion of increased health awareness by men round the age of 40 may indicate a window of opportunity to intervene at this time.

## Background

The 2007 Wanless report [1] highlights the ever increasing problem of obesity and the consequent health problems. Despite many attempts to develop interventions to prevent obesity, little success has been reported. Levels of obesity in the UK are predicted to rise to 33% of men, 28% of women and over 20% of all children by 2010. The health consequences are well documented; for example obesity is estimated to account for a 54% increase in the incidence of type 2 diabetes between 1998 and 2003 [2]. Even though in 'westernised' countries there is a strong association between self perceived weight status and weight control behaviour [3], men are much less likely to perceive themselves as over weight than women across all weight categories [4,5]. Men of lower socioeconomic status are known to have higher levels of abdominal obesity, be at greater risk of cardiovascular disease (CVD) and diabetes and be particularly hard to reach with health promoting interventions. Too little is known about middle-aged men's attitudes to and understanding of obesity, diabetes and CVD.

An increasing evidence base suggests that in terms of reducing diabetes and CVD risk it is better to be "fit and fat" than unfit and of normal weight [6]. There is evidence to suggest that much of the morbidity and mortality associated with overweight and obesity is due to being physically inactive. It is suggested that messages which focus on weight loss alone are unsuccessful and that a more successful prevention strategy may result from finding ways of increasing physical activity. In this qualitative study we examine the views of men on the subjects of weight, diet and exercise.

## Methods

This was a qualitative study in which a purposive sample of men (stratified for age, BMI, and post code) aged between 25 and 45 years was recruited from the patient register of one general practice list in Newcastle upon Tyne UK. Men who responded to a letter of invitation took part in a one to one audio-recorded semi structured interview with LH at a location of their choice. No incentives were offered to participants to take part. An interview schedule developed by the joint research team (based on a previous study [7]), used questions to stimulate discussion around topics of interest (i.e. overweight and obesity; diet; physical activity; diabetes), rather than to elicit specific answers (Table 1). BMI was recorded for each participant. Recruitment ceased when saturation of themes was achieved, that is, when no new themes emerged. Interviews were transcribed for analysis, and coded for anonymity. Initial thematic analysis of data was carried out separately by both NW and LH using framework analysis [8]. All text was systematically coded for subject matter. Emerging themes were examined by the project team. Further detailed analysis was conducted by NW and MM. Analysis was an iterative process in which the process of writing itself informed understanding [9]. Emerging themes were interlinked, and we found striking similarities in the views and perceptions of the men in the study-presented below.

**Table 1:** Interview schedule

I wonder if you could tell me about why you agreed to take part in this study.

**Overweight and obesity**
Weight problems and obesity are in the news a lot.
• I wonder if you could tell me what you think obesity means. How would you explain it to someone?
• How would you define a normal/healthy weight? (Are they the same thing?)
• What do you think influences peoples views about this?

**Diet**
• What would you describe as a healthy diet?
• Does the diet you normally eat differ from this-if so in what way?
• What do you think influences people in the choices they make about what
they eat?

**Physical activity**
• What do you regard as physical activity?
• How important do you think it is? (-views on frequency, time, and amount of exertion/-is it walking, running, housework?)
• Is it equally important for everyone- are there groups of people who should or shouldn't do much exercise?

**Diabetes**
• Do you know what diabetes is? How would you describe it to someone else? (causes/symptoms/how do people get it/who gets it/do you think is it becoming more or less common/why might that be?)
• Do you know if diabetes is linked to any other health conditions?
• Do you know anything about the ways in which diabetes can be prevented?

**Conclusion**
Are there things that we have not discussed but which you would have wanted us to discuss?

The participants are referred to using pseudonyms (see table 2). For longer quotations see tables 3, 4, 5, 6.

**Table 2:** Participants

	'Name'	Profession	Education	Age	M/S/D	Height cm	Weight kg	BMI	Waist	Hip Cm
1	Dave	Taxi driver	O level	44	M	164	84	31	105	99
2	John	Technical Manager	Degree	39	S	178	96	30	100	97
3	Mark	Self employed. Haulage contractor	A level	41	M	178	106.1	33	118	105
4	Rob	Engineering machinist	O level	44	M	162.5	83.4	32	103	100
5	Jim	IT manager	Degree	37	M	177	78.5	25		
6	Chris	Finance manager	A level	34	S	182	89.1	27		
7	Mat	Accountant	FE	40	D	183.5	95.1	28		
8	Peter	Quantity surveyor	FE	37	M	176.5	106.9	34	112	107
9	Craig	Trainee solicitor	Degree	36	S	168	70.9	25	79	86.5
10	Mike	Civil servant (manager)	A level	34	M	173	81	27		
11	Ben	Insurance (manager)	Degree	44	M	177.5	74	24	88	94
12	Steve	Canvasser (double glazing)	No qual-ifications	40	D	168	100.5	36		
13	Jack	Rehabilitation engineer	FE	34	M	178	104.2	33	96	108
14	George	Investment manager	Post graduate degree	31	M	186	76	22		
15	Richard	Factory worker	FE	35	M	187	98.5	28	105	104
16	Nigel	Council (manager)	Degree	35	M	169.5	80.5	28	93	99
17	Andy	Postman	O level	28	S	175	84.1	27	93	106

M = married, S = single, D = divorced, FE = further education unspecified.

**Table 3:** (Quotes)

**Obesity and excess weight**
1.1 "I have always had this sort of stigma, obese people being sort of round shapes with legs" (Mark, line 35–36); and, "Very large waist, you know, very, very big arms at the top, not so wide at the bottom, just looks oversized or inflated, somebody whose general appearance looked like an inflated balloon". (Jim, line 39–41).

1.2 "to me obese is, is not what I am [BMI 32], its basically these people who are a lot larger and basically, fat, I mean where you can actually see physically not a lot of muscle, so stocky and things like that, so to me obesity is that." (Jack, line 18–21)

1.3 "but I think it is somebody who maybe is too overweight to do any sort of normal physical exercise and by that would mean, if you are too overweight that you can't run or jog" (George, line 31–33)

1.4 "I mean, I am overweight, but I wouldn't say I was overweight you know, ...(Chris, line 39) I probably look better than I did a few years ago when I was less weighty you know, because I have filled out etc you know, because I mean for years I was always 11 1/2 stone, until maybe about 5 year ago and then I just you know, the thirties start and I just went "poof", so either like you know I see myself much nicer ..." (Chris, line 53–57)

1.5 "...I suppose maybe it's not directly answering the question. What I don't think is a normal look, is when people strive to be stick thin and, well it's slightly different for men, but for women, strive to be a size, sort of two sizes smaller than what they actually should be... " (Peter, line 39–42)

**Table 4:** (Quotes)

**Being fit and eating a healthy diet**
2.1 "I think everybody benefits from exercise, some form of exercise along the way". (John, line 179–80). (Referring to his arthritis): "but I found when I do exercise, it actually helps, I seem to be more alert, I seem to be more, sort of, not as stiff" (John, line 147–148)

2.2 "I think in particular if perhaps your diet is not as healthy as what it could be, I think you do need to try and balance things up a little bit..." (Mike line 187–188)

2.3 "... 5 fruit and veg. a day, brown bread, brown rice...Stay clear of chocolate, fats, high salt..." (Rob line 91–2, 94)

2.4 "much more in the press and sort of the Government coming out with you have got to eat 5 vegetables a day or whatever and the sort of food labelling" (George, line 108–110)

2.5 "there is always fresh fruit in the house and things like that, I try and keep away from microwave meals (Jack line 71–72)... we don't eat a lot of fried food you know, if everything is done, it would be grilled or it would be boiled" (Jack line 78–79)

2.6 "a lot of it could be social aspects. I mean those on a basic, the old bread line or less wages will have to I mean, it's always the cheap sugary foods that are cheaper. I mean, ourselves we have notice even when trying to buy a load of fruit and veg, I mean, its very expensive compared to, you can go and buy something else, buy the ingredients, sometimes it is just as easy to buy the other stuff. So, I mean...I think there is a lot of economic influences in it..." (Jack line 83–90)

2.7 "there's huge cost to exercising properly if you wanted to join a club with membership, and I think you know, there isn't a lot of places where you could go running safely when you get home, (Nigel line 291–293)

2.8 "and I went to work in a Chinese takeaway as a delivery driver, so I used to get given food by them and I wasn't eating healthily I was eating takeaways for my dinner, having tea in my house and having something when I was out working, and it was just very unhealthy" (Richard line 119–123)

2.9 "...their lifestyles, yes I would say, I know it is being pretty awful and general, but if you are sat in a cab for 4 or 5 hours a day, and you just park up, walk across to the café, motorway cafes, I mean I know myself that for £3 you can have bacon, double sausage, mushroom and blah, blah, blah with chips, not the best diet in the world" (Mark line 311–315)

2.10 "I seen it as an opportunity to get myself fitter, and then when I did get put on the production line" (Richard line 74–5).

**Table 5:** (Quotes)

**Age and change**
3.1 My work has changed, also with having the kids, what I used to do changed. I used to go running every night... (Mark line 85–6)

3.2 ...the kids, he's nearly 15 and my daughter is 13, so I would rather be here and be able to do things with them, you know I mean, when they are 25, I don't want to sitting there going, oh go on I can't come with you, you know, that's the thing being in life is like, I need to be there for them Dave (line 171–174)

3.3 "I think if you came in and your mum and dad were just flopped on the settee doing nothing, well you would just flop next to them wouldn't you, so..." (Ben line 90–92)

3.4 "I played football until my knees went, so I have always been active, sportingly, not so much recently because of the demands of my job, but we do try and get out at weekends for walks and that sort of thing, go swimming, like I said I play golf when I can, so I try and keep active, I am not completely sedentary, but I know I could do more as well." Rob (line 119–123)

3.5 "Well, myself, it's a lifestyle change. Up until say, a few years ago, I went to the gym regularly, that was probably 3 times a week for an hour, two hours after work, sort of thing. I also did a lot of mountain biking, but then you know, one day you put your bike away and you say I'll get back on it, and one day you don't go to the gym and you say I'll got back tomorrow, you just fall out of your routine... (Chris line 82–9)

3.6 "...is it the fact that you are getting older and if someone when you were 20 said, your uncle has got diabetes, within two seconds you would have forgotten that it happened, but because now its developed ... that maybe you are just more aware of it." (Ben line 214–18)

3.7 "...sometimes, especially since I have got older like, obviously I am 39 now. I think up to about the age of sort of late 20's, I classed myself as okay (John line 63–4)... I got to a certain age and thought oh right I am starting to put the weight on here a bit, I'll start doing a bit of exercise and start doing, sorry, eating a little bit more healthier than I used to" (John line 82–5) [on diabetes] "...no, no I definitely hear more about it now. I don't know if it is just because of the age group of the people I am in like, younger, maybe they didn't have it and its just maybe it's developing it now, see with the onset of age and that. (John line 243–5)

**Table 6:** (Quotes)

**Diabetes**
4.1 "I know very little about diabetes I mean you know, I've heard...well I've known people in the past that had diabetes who were insulin dependent they had to obviously take medication to control that, and you know, I've worked with those people and they were able to ...signs and symptoms and know when they were needed to either take sugar or whatever, diabetes that great, and obviously when my father has it he doesn't...he takes medication to control his, but quite active and quite healthy still, it's under his control," (Nigel line 307–14)

4.2 "It was the diabetes part as well (6 line 5)...in as much as it's not pushed in your face. You know obesity is touted every day on the 'tele' and on the internet, there is always some health study showing this, that and the other you know, and they always tend to, I would say concentrate a lot on high blood pressure and so forth, in fat people, obese people or who are overweight you know, and so when I was reading it out to my other half, I said to her, I don't really know much about the health implications or anything about obesity apart from the usual, you know, the blood pressure factor." (Chris line 11–18))

4.3 "I have got a friend, a friend and neighbour who suffers from diabetes and he is the same as myself, overweight in some respects so I thought, well..." (Mark line 6–8

4.4 "you know the people who are clinically obese and whatever, diabetes always seems to be mentioned in there as well, so but I don't know the direct link..." (Ben line 230–2)

4.5 "If you are overweight you are [more at risk of diabetes] but I think that is generally a lifestyle type of thing, because to get overweight you have obviously not been eating or drinking healthily" (George line 238–40)

4.6 "I know that it affects other organs, but I can't, as I said to you I can't sort of think of an example" (Peter line 138–9)

4.7 "I am sure my dads been told he's more likely to have a heart attack because of this which is wonderful because he is high risk anyway, just another thing that you can pass onto us. (George line 273–5)

4.8 " [on excess weight] Heart attacks, blocking of the veins, diabetes, muscle aches, muscle pains, everything. Once you have too much fat in the bloodstream, that's it". (Steve line 141–2)

4.9 "eyesight isn't it that can deteriorate, and well obviously if you have got weight problems, if you are carrying weight, there is arthritis in your knees, heart problems, that kind of thing." (Craig line 120–22)

### Ethical and research governance

Approval for the study was given by Northumberland Local Research Ethics Committee and Newcastle PCT.

## Results

One hundred and forty invitation letters generated 17 positive responses. The 17 men interviewed (table 2) were aged between 28 and 44, with more participants towards the older end of the invited group (one aged < 30 yrs; four aged 30–34 yrs; six aged 35–39 yrs; six aged 40–45). Participants had a broad spread of BMI values (BMI 25 or less = 4; BMI 26–29 = 6; BMI 30–35 = 6; BMI of 36 = 1). The men in the study had a variety of educational background and occupation. None were unemployed. 9/17 participants had 'A' levels or degree, and a further 3/17 had undertaken some form of further education. Below we present the views and perspectives which emerged from the thematic analysis of the data.

### 1. Obesity

It was clearly stated that when the word obesity is used in everyday language it does not have the same meaning as when it is used in a clinical setting:

"...there is a difference between the common opinion of what obesity is, and what clinical obesity is..." (Jim, line 15–16). Obese people were described by participants as being fat with an obvious central distribution of the excess weight. Words such as "round" (Mark 1.1) and "balloon" are used (Jim 1.1). Having a lack of obvious muscle is seen as part of the definition of obesity by some (Jack 1.2), and again when Dave (line 64) describes his appearance he points out that he has "muscly arms and legs" giving the impression that this somehow counterbalances his excess weight. Ideas about obesity incorporated an element of difficulty in taking exercise in the definition, so for Richard (line 37–38) obesity is "Somebody being overweight, and not necessarily unfit, but struggle with their breathing and things like that, and struggle to move around" Here obesity is described in the sense of bodily experience and of leading to health problems rather than as being a health problem per se.

Many (but not all) men who had a BMI greater than 25 described feeling comfortable with how they look, and felt that the extra weight was not bad for them if they led an otherwise healthy lifestyle e.g. Chris (1.4) BMI 27. Peter (1.5) with a BMI of 36 thinks it is not normal to be too thin and talked about the harm caused, particularly to women, by striving to be "two sizes smaller than what they actually should be". For Peter to be too thin is seen as harmful whereas to be overweight is not described as such. Men demonstrated clear recognition that health problems can result from being very heavy but they also described people with a high BMI who are active strong people who appear healthy and fit, rather than the round shape that is recognised as obese. There was evident understanding that people can have a high BMI as a result of muscle mass, as well as because of excess fat. In addition there was understanding of the complexity of issues relating to weight and fitness. It was recognised that people of the same weight, normal or otherwise, can have very different fitness levels and are therefore very different in terms of health. Men placed a high value on physical fitness and interlinked the issue of weight with this, placing less importance on any fixed idea of a correct weight.

### 2. Being fit and eating a healthy diet

Many of the men described having taken part in regular sporting activity at some point. Men valued exercise in a variety of ways. Some like the way it makes them look; others value the weight control aspect. Others simply value it for its effects on well being (John 2.1), or as a sociable activity. Mike (2.2) believes that there is a balance to be struck whereby exercise can counteract some of the ill effects of an unhealthy diet.

There was good knowledge of what constitutes a healthy diet, and many men would prefer to eat this way at least part of the time. There was awareness of Government intervention in promoting public health messages around diet, and of the influence of the media. Most men could give a brief description of a sensible approach to healthy eating, mentioning five pieces of fruit and vegetables a day, (Rob 2.3 and George 2.4), and including references to brown bread, brown rice, avoiding fats and high salt, avoiding fried and processed foods (Jack 2.5). Others describe their approach to buying and cooking food in a health conscious way.

Employment was seen as a key factor in the perceived restrictions on both eating a healthy diet and opportunities to exercise. Access to food was discussed in a number of ways. The time pressure created by long work hours makes it difficult for people to think about what they are eating, as well as difficult for them to get to the shops. Another access issue related to the higher cost of healthy food. Jack (2.6) and Nigel (2.7) pointed out that economic factors push the less well off into a less healthy life style. Free food available to (usually low paid) fast food workers (e.g. Richard 2.8) is a significant financial benefit. Jobs in which men are 'out and about' driving all day make access to cheap poor quality food easy (Mark 2.9), compounding the fact that these jobs (e.g. taxi driving) are sedentary and create little opportunity for exercise. Others, who are 'tied' to a desk, have similarly limited opportunities to exercise during the day. For some however work patterns keep them fit. Steve works as a double glazing "canvasser" and walks 5–6 miles per day. Richard (2.10) works on the production line at a local factory and values the physical fitness that results from this.

### 3. The changes that come with age

Men in this study talked in many ways about the changes occurring in their lives and how these affect them. They have noticed changes in their own levels of awareness of health and illness, usually because of personal contact with colleagues, friends or family experiencing ill health.

Change often results in increasing complexity and competing demands on time. Children have a major impact-seen by some as an obstacle to a healthy lifestyle (Mark 3.1), and by some as the opposite Dave (3.2), (Ben 3.3).

New health issues, particularly the onset of knee problems were described as preventing a number of men from taking part in sports which they previously enjoyed. "I played football until my knees went" (Rob 3.4) There was much discussion about the negative impact knee injuries have had on fitness opportunities, but only one man mentioned the impact that excess body weight has on causing and aggravating knee problems.

For some men all of this has resulted in loss of routine (Chris 3.5) and decreased physical activity. For others, particularly those in their late 30s and early 40s, there seems to be an increased awareness of health as a vulnerable condition, and of the importance of a healthy lifestyle. Ben (3.6), 44 yrs old, described being more conscious of health related issues than when he was younger, now noticing when people around him develop health problems. John (3.7) has noticed that he comes across people with diabetes more often. He relates this, and the fact that he is paying more attention to diet and exercise, to his age.

### 4. Diabetes

Many of the men interviewed were aware of diabetes because someone they know has the condition. Most men do not think that they understand much about diabetes – they know that there is more than one type of diabetes but they don't understand how the different types fit together. Nigel (4.1) stated that he doesn't understand diabetes but has observed that in some situations diabetics need to eat sugar to relieve symptoms. His father is diabetic but apparently doesn't need to take treatment and is well and active. He finds this confusing. Similarly Mark (line 248–9) was aware of a degree of confusion on the subject-he understands that there are two types of diabetes, which he describes as very different "both the opposite case".

The profile of the condition was seen as less prominent than some other health problems. Chris (4.2) is aware that he is picking up information about other health conditions from the media but has noticed less information about diabetes. Some are aware of the link between diabetes and excess weight e.g. Mark (4.3), who was recently shocked to be classed as obese by his doctor. Many were not clear about the significance of excess weight however. Ben (4.4) wonders if weight is just a marker for an unhealthy lifestyle. George (4.5) describes the association of weight and diabetes but wonders if diabetes results from the unhealthy lifestyle underlying the weight problem.

Some men were aware that eyesight can be damaged by diabetes; cataracts glaucoma and bleeding were mentioned. There was limited awareness of what other problems result from diabetes. Peter (4.6) understood that diabetes is a significant condition but did not have a clear idea about what that implies in terms of illness. George (4.7) had heard that heart problems can result. Steve (4.8) describes the problem of obesity as causing "too much fat in the blood stream" but an equivalent understanding of the effects of too much sugar in the blood stream was not as evident. Craig (4.9) made the link between excess weight and heart problems, but without knowing that diabetes is an added risk factor.

## Discussion

The main findings of this study are as follows:

• The men in this study understood the word obesity differently from the clinical definition; the term "obesity" was used mainly as a description of those with centrally distributed fat, and understandings of the term commonly took into account fitness as well as weight.

• Men described becoming more aware of health issues in theirlate 30s and early 40s, suggesting there may be a window of opportunity to intervene at this time.

• Knowledge of healthy lifestyles was generally good, but men described difficulty acting upon this knowledge as they approach middle-age and have increasing responsibilities at home and at work.

• Knowledge of diabetes and the link between obesity and diabetes was poor.

This study set out to understand important health issues from the individual perspectives of men in the general population. As a qualitative study this research sets out to understand the *range *of views held by men but does not make claims about the *distribution *of those views, and is therefore not generalisable in the quantitative sense. We can however generalise theoretically in the sense that the understandings gained here enable us to better judge the issues of importance to men in this age and socio economic group and provide the parameters for quantitative studies of the distribution of these understandings. Validity in qualitative research is established through standard mechanisms to assess the plausibility and credibility of the claims made [10,11]. Here this was achieved through saturation of themes, exposition of methods, attention to negative cases and reflexivity. While we interviewed men in the age group 25 to 45 years and achieved a good mix of weight and occupation, the group had higher levels of educational attainment than would be expected in the general population and this is a potential source of bias. Our conclusion that men in their late 30s and early 40s are open to change could result from having particularly attracted a group of participants who feel this way. Conversely however, the lack of understanding of diabetes in this relatively well educated group suggests that the problem may be much worse in a different sample which better represents the average educational achievement in the population at large.

For men in this study the word obesity implies elements relating to the central distribution of body fat, and to levels of physical fitness. The focus on 'round' body shape interestingly fits with scientific research which suggests that waist circumference is a better measure of cardiovascular risk that BMI [12]. Many men actually prefer the way they look when their BMI is > 25 and do not see this higher level of weight as a problem in the context of a healthy lifestyle. These men aspire to be physically fit, and public health messages about healthy eating seem to have made an impact. They discuss the factors which limit opportunities for exercise and healthy diet. Patterns of employment are seen as very significant, as well as the related economic factors e.g. sedentary occupations, such as taxi driving, and easy access to cheap fast food. They notice an age related increase in their awareness of health issues.

The ideas we demonstrate about the scepticism held in relation to a target BMI of < 25 have been seen elsewhere. Monaghan interviewed 37 men, (mean age 43), exploring the way men felt about conforming to a 'healthy' BMI, and found "talk about the compatibility of heaviness, healthiness and physical fitness; looking and feeling ill at a putatively 'healthy' BMI; and the irrationality of standardisation" [13].

Research showing that the association between overweight or obesity and mortality is markedly attenuated and in some instances eliminated when objectively measured cardio respiratory fitness is included in the statistical models [14] fits with the perception of the men in this study, that any level of weight cannot be viewed in isolation from levels of physical fitness.

For the men in this study the word diabetes conjures up a number ofconcepts which link to it, but are not easy to understand as a whole. Facts relating to 'type one' and 'type two' diabetes are mixed together, and the side effects of insulin are mixed up with the signs and symptoms of diabetes. Diabetes is understood as an illness, rather than a risk factor, but people are confused about the lack of tangible symptoms relating to the 'illness' of diabetes itself. The understanding of diabetes is like self assembly furniture without the instructions-there are lots of pieces which clearly go together but it is not clear how.

There is little published research into the general population's understanding of diabetes with which to compare these findings. Diabetes UK commissioned a MORI conducted survey of 2,135 adults in the UK [15] in 2000 which found that public understanding of diabetes and its impact is poor. Four out of five people believed that some people get a milder form of diabetes than others; three quarters (76 per cent) of those in high risk groups were unaware of their risk of developing the condition; less than half (46 per cent) of the public know that death can result from diabetes; only one quarter of the population know that diabetes can lead to heart disease despite the fact that it significantly increases the risk. Our findings mirror these results.

A number of men described knee pain as having significantly interrupted their exercise regime. The perception seems to be that exercise is responsible for the problem, and that weight gain is a consequence. Some evidence suggests that participation in exercise does not cause knee osteoarthritis [16], but other more specific follow up studies of professional footballers do indicate long term problems, including osteoarthritis of the knee [17]. Obesity however is clearly associated with knee pain and osteoarthritis in the knee [18]. In obesity and diabetes prevention in men, education about good knee care, and the impact of weight on knee problems may be an important factor.

## Conclusion

We illustrate that middle aged men have a complex understanding of what it is to lead a healthy lifestyle, which includes the interlinked nature of weight and fitness. Obesity is understood as a description of people with centrally distributed fat, in association with low fitness levels, rather than simply a function of raised BMI. In general, it seems that limitations on ability to adhere to a healthy lifestyle relate less to lack of understanding of the issues than to limiting economic, social and physical factors. Raised levels of awareness may mean that there is an opportunity for intervention with men around the age of 40 to address factors affecting weight maintenance and participation in exercise. Understanding of the condition of diabetes stands out as being confused and there is a need to improve the general understanding of this condition.

## Competing interests

The authors declare that they have no competing interests.

## Authors' contributions

LH and NW carried out the recruitment of participants. LH arranged and carried out the interviews. Initial data analysis was done by NW and LH. NW performed further data analysis and manuscript preparation. MM and NU participated in regular meetings to scrutinise the analysis process and conclusions. All authors read and approved the final manuscript.

## Pre-publication history

The pre-publication history for this paper can be accessed here:


